# Specific Integration of Temperate Phage Decreases the Pathogenicity of Host Bacteria

**DOI:** 10.3389/fcimb.2020.00014

**Published:** 2020-02-04

**Authors:** Yibao Chen, Lan Yang, Dan Yang, Jiaoyang Song, Can Wang, Erchao Sun, Changqin Gu, Huanchun Chen, Yigang Tong, Pan Tao, Bin Wu

**Affiliations:** ^1^State Key Laboratory of Agricultural Microbiology, College of Veterinary Medicine, Huazhong Agricultural University, Wuhan, China; ^2^The Cooperative Innovation Center for Sustainable Pig Production, Huazhong Agricultural University, Wuhan, China; ^3^Beijing Advanced Innovation Center for Soft Matter Science and Engineering (BAIC-SM), College of Life Science and Technology, Beijing University of Chemical Technology, Beijing, China; ^4^Division of Pathology, College of Veterinary Medicine, Huazhong Agricultural University, Wuhan, China

**Keywords:** *Bordetella bronchiseptica*, temperate phage, bacterial virulence, integration site, attenuation

## Abstract

Temperate phages are considered as natural vectors for gene transmission among bacteria due to the ability to integrate their genomes into a host chromosome, therefore, affect the fitness and phenotype of host bacteria. Many virulence genes of pathogenic bacteria were identified in temperate phage genomes, supporting the concept that temperate phages play important roles in increasing the bacterial pathogenicity through delivery of the virulence genes. However, little is known about the roles of temperate phages in attenuation of bacterial virulence. Here, we report a novel *Bordetella bronchiseptica* temperate phage, vB_BbrS_PHB09 (PHB09), which has a 42,129-bp dsDNA genome with a G+C content of 62.8%. Phylogenetic analysis based on large terminase subunit indicated that phage PHB09 represented a new member of the family Siphoviridae. The genome of PHB09 contains genes encoding lysogen-associated proteins, including integrase and cI protein. The integration site of PHB09 is specifically located within a pilin gene of *B. bronchiseptica*. Importantly, we found that the integration of phage PHB09 significantly decreased the virulence of parental strain *B. bronchiseptica* Bb01 in mice, most likely through disruption the expression of pilin gene. Moreover, a single shot of the prophage bearing *B. bronchiseptica* strain completely protected mice against lethal challenge with wild-type virulent *B. bronchiseptica*, indicating the vaccine potential of lysogenized strain. Our findings not only indicate the complicated roles of temperate phages in bacterial virulence other than simple delivery of virulent genes but also provide a potential strategy for developing bacterial vaccines.

## Introduction

Bacteriophages (or phages), which specifically infect bacteria, are the most abundant organisms on the earth. Based on their life cycles, phages can be classified as virulent and temperate phages. Upon infection, the virulent phages take over the machinery of the host cell to produce and finally lyse the host cell to release their progenies. In addition to the lytic cycle, temperate phages also have a lysogenic cycle, in which phages incorporate their genomes into a host chromosome (or maintain their genome extrachromosomally) and replicate with it without lysing their host cells (Howard-Varona et al., 2017). Therefore, temperate phages are considered as natural vectors for gene transmission among bacteria and play important roles in virulence of bacterial pathogens (Boyd, 2012; Cuenca Mdel et al., 2016).

Many studies have indicated that the infection of temperate phages might lead to the enhancement of host virulence as many virulent genes of pathogenic bacteria were identified in the phage genome (Boyd, 2012). For instance, lambdoid phages encode both subunits, *Stx*1 and *Stx*2, of Shiga toxins, which are the major virulent factor of Shiga toxin-producing *Escherichia coli* (Herold et al., 2004). Many effector proteins, which are bacterial virulent factors and are injected into host cells by bacterial type III secretion system to help bacterial invasion and survival, were identified in *Salmonella typhimurium* phages (Figueroa-Bossi et al., 2001). Infection with such temperate phages might lead to the transmission of the virulent genes, thus increases the virulence of host bacteria (Cuenca Mdel et al., 2016). Additionally, it was reported that temperate *staphylococcal* phage 80α could efficiently encapsidate and transfer bacterial pathogenicity island, SaPI1, to a recipient strain (Ruzin et al., 2001). In contrast to the enhancement of virulence, however, the roles of temperate phages in attenuation of bacterial virulence are largely unknown.

*Bordetella bronchiseptica* is a common pathogen colonizing the upper respiratory tract of a variety of animals (Goodnow, 1980). Meanwhile, it was reported that *B. bronchiseptica* can cause severe pulmonary infections in immunosuppressed patients, such as people with lymphoproliferative disorders (Stoll et al., 1981), HIV infection (Rampelotto et al., 2016), cystic fibrosis (Register et al., 2012), and lung transplantation patients (Ner et al., 2003). In addition, *B. bronchiseptica* infection causes atrophic rhinitis and bronchopneumonia in pigs, leading to a huge loss to the pig industry (Brockmeier, 2004; Brockmeier et al., 2008). Recently, drug-resistant *B. bronchiseptica* strains were frequently reported, which brings an urgency of developing new strategies to treat or prevent the infection (Kadlec et al., 2004; Zhao et al., 2011; Pruller et al., 2015a). Phage therapy could be an alternative strategy against drug-resistant *B. bronchiseptica* infection. In fact, several *B. bronchiseptica* phages have been isolated (Liu et al., 2004; Petrovic et al., 2017; Chen et al., 2019a), and some of those were tested against *B. bronchiseptica* infection in our previous study (Chen et al., 2019a).

We have been working on isolation and characterization of *B. bronchiseptica* phages since we reported the use of phages to treat *B. bronchiseptica* infection (Chen et al., 2019a). An interesting temperate phage, PHB09, which has a specific integration site in the host genome, was found from our *B. bronchiseptica* phage collections. The aim of this study was to investigate the effects of PHB09 integration on its host. Interestingly, we found that the specific integration of PHB09 significant decreased the virulence of parental strain *B. bronchiseptica* Bb01 in mice. Furthermore, mice intranasally inoculated with 1.8 × 10^8^ colony-forming unit (CFU) lysogenic strain were completely protected when challenged with 3.0 × 10^8^ CFU *B. bronchiseptica* Bb01, indicating the vaccine potential of the lysogenic strain. To our knowledge, this is the first report that the integration of temperate phage reduces the virulence of host bacteria in the mammal, indicating the complicated roles of temperate phages in bacterial virulence other than the simple delivery of virulent genes. Our study also provides a new strategy to design avirulent strains for developing bacterial vaccines.

## Materials and Methods

### Bacteria

Totally, 119 *B. bronchiseptica* strains were used in the current study. These strains were isolated from pig lung secretions between 2016 and 2018, and confirmed by PCR using *B. bronchiseptica*-specific primers, Fla-fw (5′-CCCCCGCACATTTCCGAACTTC-3′ and Fla-rw (5′-AGGCTCCCAAGAGAGAAAGGCTT-3′) (Hozbor et al., 1999). *Escherichia coli* DH5α and two *Pasteurella multocida* strains (capsular type A and D, respectively) were used to determine the host specificity of phage PHB09. *E. coli* ATCC 25922 was used as a control strain for antimicrobial susceptibility testing. *B. bronchiseptica* and *P. multocida* were cultured at 37°C in tryptic soy broth (TSB; Becton Dickinson, NJ, USA) or on tryptic soy agar (TSA; Becton Dickinson, NJ, USA) supplemented with 10% (v/v) sterile defibrinated sheep blood (Jiulongbio, Zhengzhou, China). *E. coli* strains were cultured in TSB/TSA medium at 37°C. All bacterial strains are available from the State Key Laboratory of Agricultural Microbiology, Huazhong Agricultural University, China.

### Phage Isolation and Purification

Phage isolation and purification were carried out using a double-layer agar method as described previously (Chen et al., 2019a). Briefly, sewage samples (20 ml each) were collected in Jingzhou, China, and sterilized by filtration through a 0.22-μm membrane. Five milliliters of each filtrate was added to 10 ml of exponential-phase *B. bronchiseptica* Bb01 culture, respectively. After 12 h incubation at 37°C, the cultures were centrifuged at 12,000 g for 10 min, and 300 μl supernatant of each sample was mixed with 500 μl of *B. bronchiseptica* Bb01 in the 15-ml tube. After adding 6 ml of molten soft TSA (0.75% agar) supplemented with 10% sterile defibrinated sheep blood, the mixtures were poured into prepared TSA plates and incubated at 37°C overnights. The single plaques were picked up individually and inoculated into 5 ml of log-phase *B. bronchiseptica* Bb01 culture. After 12 h incubation at 37°C, the cultures were filtered through 0.22-μm membranes to remove bacterial cells. The filtrate containing phages was titrated, and the phages were subject one more round of purification. For electron microscopy analysis, phages were purified by CsCl gradient ultracentrifugation as described previously (Thomas et al., 2007; Tao et al., 2016, 2017). Phages were placed on carbon-coated grids, washed gently with a few drops of distilled water, and stained with 2% uranyl acetate. The stained phages were observed under a 100-kV transmission electron microscope (HITACHI H-7650, Japan).

### Genome Characterization of Phage PHB09

Genomic DNA of phage PHB09 was extracted using phenol-chloroform as described previously (Chen et al., 2019b) and dissolved in TE buffer (10 mM Tris-HCl [pH 8.0], 1 mM EDTA). The genomic DNAs were sequenced using the Illumina MiSeq system (San Diego, CA, USA) and assembled using Newbler software v3.0. Putative open reading frames (ORFs) were predicted using RAST (Aziz et al., 2008; Overbeek et al., 2014; Brettin et al., 2015). The assembled genome sequence was searched against protein and nucleotide databases using the basic local alignment search tool (BLAST) (http://www.ncbi.nlm.nih.gov/BLAST/). Protein BLAST (BLASTP) was used to identify proteins sharing similarities with the predicted phage proteins. The tRNA genes were predicted using tRNAscan-SE (Lowe and Chan, 2016; Chan and Lowe, 2019). Promoters and terminators were analyzed using the BPROM (Solovyev and Salamov, 2011). Phylogenetic tree of the phage large terminase subunit sequences was generated using MEGA 6 with a bootstrap re-sampling analysis of 1,000 replications (Tamura et al., 2013).

### Determination of Phage PHB09 Host Range

Totally, 119 *B. bronchiseptica* strains, as well as two *P. multocida* strains (capsular type A and type D, respectively) and *E. coli* DH5α, were used to determine the host range of phage PHB09 using the double-layer agar method as described above. Briefly, 300 μl of the exponential-phase culture of each strain was mixed with 6 ml of molten soft TSA (0.75% agar) individually and plated into prepared TSA plates. Five microliters of SM buffer (0.1 M NaCl, 8 mM MgSO_4_, 50 mM Tris-HCl [pH7.4], and 0.01% gelatin) containing 5 × 10^5^ PFU phages was spread on top of the soft agar. The plates were incubated at 37°C overnight and observed for the presence of plaques.

### Screening for Strains Lysogenized by PHB09

To identify strains lysogenized by PHB09, 100 μl of exponential-phase *B. bronchiseptica* Bb01 culture (~10^8^ CFU/ml) were incubated with 100 μl of purified phage PHB09 (10^9^ PFU/ml) as previously described (CLSI, 2018; Chen et al., 2019a). Six milliliters soft TSA (0.75% agar) containing 10% fetal bovine serum was then added to the mixture and then poured onto a prepared TSA plate. After 12 h incubation at 37°C, the phage-resistant colonies were picked and inoculated into 10 ml TSB medium containing 10% fetal bovine serum. Subsequently, every colony was examined for the sensitivity against phage PHB09 infection by spot test using 5 × 10^5^ PFU phages. The presence of prophage PHB09 was verified by PCR amplification of phage genes, integrase (ORF1), cI (ORF14), and large terminase subunit (ORF31). The specific integration sites of phage PHB09 were determined by PCR with two sets of primers, 4F/4R and 5F/5R and Sanger sequencing of *B. bronchiseptica* genome DNA with primers 5F and 4R (Table S3).

### Growth Curve of *B. bronchiseptica*

The growth curve of *B. bronchiseptica* was carried out as described previously (Pruller et al., 2015b). Briefly, a single colony of phage PHB09 lysogenized strain, Bb01+, and the parental strain, Bb01, was individually picked up and inoculated into a 15 ml tube containing 5 ml TSB medium supplemented with 10% (v/v) sterile defibrinated sheep blood. After overnight incubation at 37°C, culture was inoculated at a ratio of 1:1,000 into 500 ml flask containing 150 ml fresh TSB medium supplemented with 10% (v/v) sterile defibrinated sheep blood and incubated at 37°C for 42 h. Samples (0.2 ml) were collected every 2 h, and titers were determined by plating 10-fold serial dilutions of samples.

### Serum Sensitivity Assay

One hundred microliters *B. bronchiseptica* cells (1.5 × 10^4^ CFU/ml) were mixed with 100 μl of mouse serum (Yisheng, Shanghai, China), inactivated mouse serum, and PBS, respectively. After 1 or 2 h incubation at 37°C, the titers of *B. bronchiseptica* were determined as described above.

### Adhesion and Invasion Assays

Human epithelial type 2 (HEp-2) cells were used to analyze the adhesion and invasion ability of strains, Bb01 and Bb01+, as described previously (Yang et al., 2016; Fu et al., 2018). Briefly, HEp-2 cells were seeded in six-well plates at the density of ~10^6^ cells per well and incubated overnight at 37°C in 5% (v/v) CO_2_ incubator. One hundred microliters exponential-phase Bb01 or Bb01+ cells (~10^8^ CFU/ml) were added to each well, and the plates were incubated at 37°C in 5% (v/v) CO_2_ incubator. For adhesion assays, the culture supernatant was removed after 2 h incubation, and the cells were washed three times with PBS and lysed with 0.5% (v/v) Triton X-100. Bacterial counts, which might contain both invaded and adhered cells, were determined by plating 10-fold serial dilutions of cell lysates. For the invasion assays, after washing with PBS, fresh DMEM medium containing 100 μg/ml ampicillin was added to each well. The plates were further incubated for 2 h. The culture medium was then removed, and cells were washed and lysed with 0.5% (v/v) Triton X-100. Bacterial counts were determined by plating 10-fold serial dilutions of cell lysates.

### Phagocytosis Assay

RAW264.7 cells were cultured in a six-well plate with RPMI 1640 medium (HyClone, USA) containing 10% (v/v) fetal bovine serum (Gibco, USA) as described previously (Carreras-Gonzalez et al., 2019). The cells were washed three times with PBS before adding exponential-phase Bb01 or Bb01+ cells at a ratio of 10:1 (*B. bronchiseptica* to RAW264.7). Plates were incubated at 37°C for 30 min in a 5% (v/v) CO_2_ incubator. The culture medium was replaced with fresh RPMI 1640 medium (HyClone, USA) supplemented with 100 μg/ml ampicillin to kill any extracellular bacteria. Plates were further incubated at 37°C for 2 h. The cells were then lysed with 0.5% (v/v) Triton X-100 after three times washing with PBS, and bacterial counts were determined by plating 10-fold serial dilutions of cell lysates.

### Antimicrobial Susceptibility Testing

The minimum inhibitory concentration (MIC) of each antimicrobial against *B. bronchiseptica* strains Bb01 and Bb01+ were determined via broth microdilution susceptibility testing according to the guidelines recommended by the Clinical and Laboratory Standards Institute (CLSI, 2018). The broth microdilution susceptibility testing of *B. bronchiseptica* was tested as described previously (Pruller et al., 2015b). Fourteen antimicrobials, ampicillin, ampicillin/sulbactam, tiamulin, tilmicosin, erythromycin, ofloxacin, ciprofloxacin, ceftiofur sodium, chloramphenicol, gentamicin, tetracycline, trimethoprim, sulfamethoxazole, and polymyxin B, were tested in our current study. *E. coli* ATCC 25922 was used as a control strain for antimicrobial susceptibility testing.

### The Virulence of *B. bronchiseptica* Strains Bb01 and Bb01+ *in vivo*

The minimum lethal dose of the *B. bronchiseptica* Bb01 was determined using three groups of 5-week-old female BALB/c mice (*n* = 3). Mice were anesthetized by intraperitoneal injection of xylazine (0.25 mg) and ketamine (1.25 mg), and then inoculated intranasally with 20 μl PBS containing 2-fold serial dilutions of *B. bronchiseptica* Bb01 starting from 1.5 × 10^8^ CFU. For infection experiments, 27 five-week-old female BALB/c mice were randomly divided into three groups (*n* = 9) and inoculated intranasally with *B. bronchiseptica* Bb01 (3.0 × 10^8^ CFU), *B. bronchiseptica* Bb01+ (3.0 × 10^8^ CFU), and PBS, respectively. Three mice from each group were sacrificed 3 days after infection, and the lung and trachea tissues were collected for histopathologic analyses. The remaining mice were monitored daily for 7 days for mortality. Survival was analyzed by Kaplan-Meier analysis with a log-rank test.

### Immunizations and Challenges

Twelve 5-week-old female BALB/c mice were randomly divided into two groups (*n* = 6). Mice in group II were immunized intranasally with 20 μl PBS containing 1.5 × 10^8^ CFU *B. bronchiseptica* Bb01+ on day 0, while mice in group I were immunized with 20 μl PBS and used as controls. The mice were then challenged intranasally with 20 μl PBS containing 3 × 10^8^ CFU *B. bronchiseptica* Bb01 on day 14. Animals were monitored twice daily for morbidity and mortality for 7 days. The survival mice were sacrificed 8 days after challenge for histopathologic analyses of lung and trachea tissues.

## Results

### Isolation and Characters of a Novel Phage, PHB09

**Figure 1:**
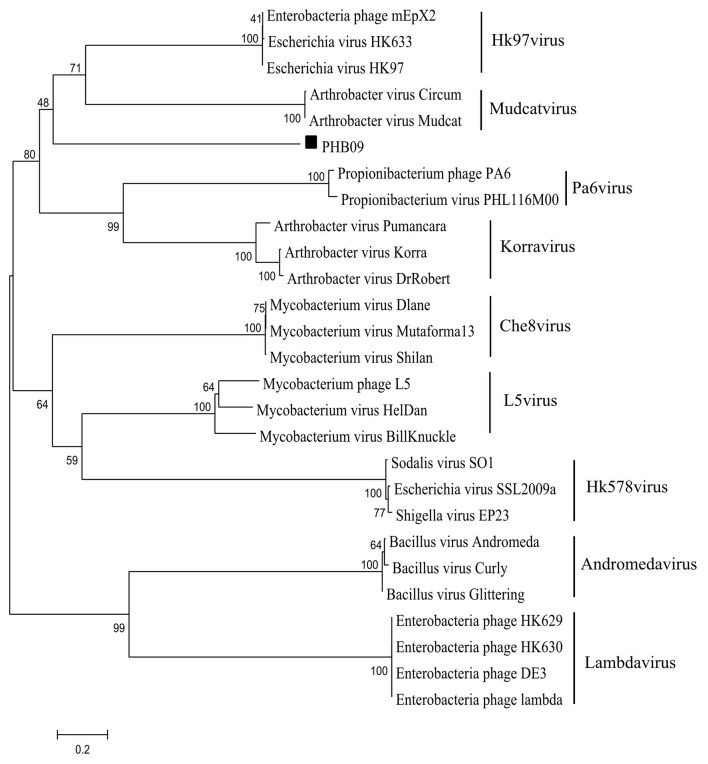
Phylogenetic analysis of phage PHB09. The phylogenetic tree was generated with MEGA 6.0 using sequences of large terminase subunit. The numbers next to the branches are bootstrap values.

**Figure 2:**
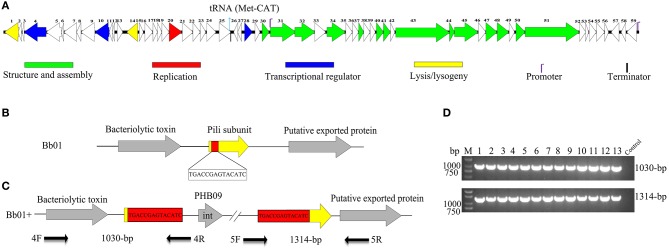
Genome organization and integration site of phage PHB09. **(A)** Genome organization of phage PHB09. ORFs are numbered consecutively from left to right. Colors denote different functional modules of genes. **(B)** The integration site of phage PHB09 in *B. bronchiseptica* Bb01 genome. The 14-bp integration site, highlighted in red, was located within pilin gene (yellow). **(C)** Analysis of the integration site of phage PHB09 in *B. bronchiseptica* Bb01+ genome. The phage PHB09 with 14-bp terminal repeat sequences was indicated. Four primers used for integration analysis and the size of corresponding PCR products were also indicated. **(D)** PCR analysis of 13 *B. bronchiseptica* strains containing prophage PHB09 with primers, 4F/4R (up panel) and 5F/5R (low panel). The *B. bronchiseptica* strain that doesn't have prophage PHB09 was used as a negative control.

After screening the sewage samples described in the materials and methods, a phage vB_BbrS_PHB09 (PHB09) was isolated using *B. bronchiseptica* strain Bb01 as a host. The initial characterization of morphology by transmission electron microscopy showed that phage PHB09 had an isometric polyhedral head and a long flexible tail (Data not shown). Based on the morphology and genome sequence, the phage PHB09 was assigned to the family *Siphoviridae*, according to the current International Committee on Taxonomy of Viruses classification system. The phage PHB09 genome was sequenced, and the large terminase subunit (ORF31) was used to generate phylogenetic trees, which also indicated that PHB09 is a new member of the family *Siphoviridae* (Figure 1). The phage PHB09 has a dsDNA genome consisting of 42,129-bp with a G+C content of 62.8% (Accession No. MN103401). The genome encodes one tRNA (Met-CAT) and 59 putative ORFs, of which 25 proteins have assigned functions and 34 are unique as no homologous proteins were identified (Figure 2A and Table S1). The entire genome contains four functional modules, structure/assembly module, replication module, transcription regulators, and lysis/lysogeny module. Genes in the structure and assembly module include small and large terminase subunit, portal protein, major capsid, and tail proteins (Figure 2A). The replication module contains homologs to known proteins, such as HNH homing endonuclease and methyltransferase. An integrase gene int, which shows 40% identity to the integrase of *Pseudomonas* phage phiAH14a, was found in the lysogeny module (Figure 2A and Table S1). In addition, the lysogeny related gene, *cI*, was also identified. No antibiotic resistance genes were found in phage the PHB09 genome when analyzed with ResFinder, a web-service used to examine the phage genome for antibiotic resistance genes (Kleinheinz et al., 2014). This is also supported by a minimum inhibitory concentration (MIC) assay of 14 antibiotics against lysogenized *B. bronchiseptica* strain Bb01+ (Table S2).

To determine the host range of phage PHB09, 119 strains of *B. bronchiseptica* isolated between 2016 and 2018 were tested using a double-layer agar method. Phage PHB09 was able to infect 89 strains (Table 1). The propagation efficiency of PHB09 in strains was different, with >10^6^ PFU/ml of progeny phages were obtained within 80 strains. Thirty strains were insensitive to PHB09 infection, of which 13 strains were found containing a PHB09 prophage as described below. Phage PHB09 cannot infect other bacteria such as *P. multocida* (capsular type A and type D) and *E. coli* DH5α (data not shown).

**Table 1:** Host range of the phage PHB09.

**Sensitive strains**						**In-sensitive strains**	
Progeny phage titers (PFU/ml)	<10^6^	10^6^	10^7^	10^8^	10^9^	With prophage	Without prophage
The number of strains	9	2	1	70	7	13	17

### The Integration Site of Phage PHB09 in *B. bronchiseptica* Is Unique

During analyzing the host range of phage PHB09, we identified 30 strains of *B. bronchiseptica* that were insensitive to phage infection. Since PHB09 is a temperate phage based on genome data (Figure 2A and Table S1), it is possible that these *B. bronchiseptica* strains might contain prophage PHB09, making them resistant against phage PHB09 infection. We, therefore, designed three sets of phage-specific primers (Table S3, Primers 1F/1R, 2F/2R, and 3F/3R) to screen the phage-insensitive strains for the presence of prophage PHB09 using PCR. We found that 13 of 30 strains contain prophage PHB09. This was further confirmed by second-generation sequencing of 3 randomly selected *B. bronchiseptica* strains. Interestingly, we found all the three sequenced genomes had a unique integration site of phage PHB09, which is a 14-bp sequence (TGACCGAGTACATC) (Figures 2B,C). To know whether the integration site of prophage PHB09 is unique in *B. bronchiseptica* genome, two sets of primers, 4F/4R and 5F/5R, were synthesized and used to screen all 13 lysogenized strains (Figures 2C,D and Table S3). If the lysogenized strains contain the same integration site, the primer sets will yield amplicons of 1,030 and 1,314-bp, respectively. Agarose gel electrophoresis analysis of PCR products showed that expected amplicons were amplified from all strains, indicating the integration site of prophage PHB09 is unique (Figures 2C,D). To exclude the possibility that phage PHB09 containing more than one integration site, we isolated the genome of all 13 strains and sequenced them with the primers, 5F and 4R, which match the ends of phage PHB09 (Figure 2C and Table S3). The sequencing results were consistent with PCR analysis, which further supported the finding that the integration site of prophage PHB09 is unique.

We then randomly picked up 40 phage-resistant colonies generated by infecting *B. bronchiseptica* strain Bb01with phage PHB09, and analyzed the integration sites by PCR and sequencing. The results showed that all these lysogenic strains had the same integration site, which further supports the unique integration of phage PHB09 (data not shown).

### The Integration of Phage PHB09 Reduces the Virulence of *B. bronchiseptica in vitro*

**Figure 3:**
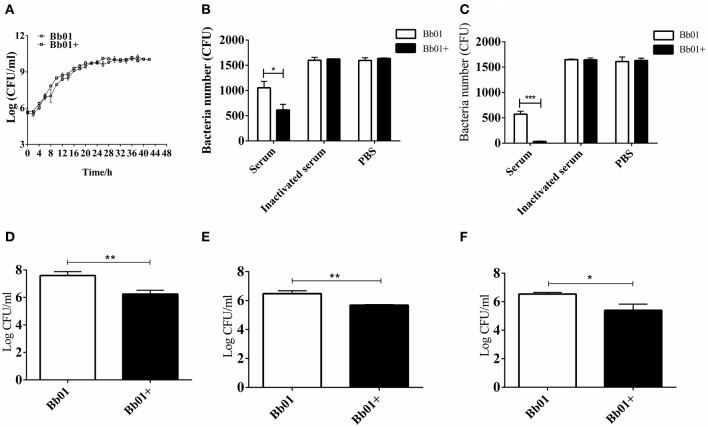
The virulence of *B. bronchiseptica* strains Bb01 and Bb01+ *in vitro*. **(A)** Growth curve of *B. bronchiseptica* strains Bb01 and Bb01+ at 37°C in TSB medium. **(B,C)** The sensitivity of strains Bb01 and Bb01+ to mouse serum. The titer of strains after 1 h **(B)** or 2 h **(C)** incubation with mouse serum at 37°C. Inactive mouse serum and PBS were used as controls. **(D,E)** Adhesion and invasion of *B. bronchiseptica* strains Bb01 and Bb01+. The same amount of Bb01 and Bb01+ cells were incubated with HEp-2 cells at 37°C for 2 h **(D)** or 4 h **(E)** respectively. After washing out the unbound bacteria, the HEp-2 cells were lysed and the bacterial counts in the lysates were determined. **(F)** Anti-phagocytic ability of the strains Bb01 and Bb01+. The titer was determined after 2 h incubation with RAW264.7 cells at 37°C. Data are expressed as the mean ± SD. *P < 0.05, **P < 0.01, and ***P < 0.001.

To explore the impact of prophage PHB09 on *B. bronchiseptica*, we firstly determined the growth of the parental strain, Bb01, and the lysogenic strain, Bb01+, in TSB medium containing serum. The results showed that the growth rate of the parental strain in the logarithmic phase was similar to the lysogenic strain (Figure 3A), indicating that the integration of phage PHB09 did not significantly affect the proliferation of the *B. bronchiseptica*. The sensitivity of strains, Bb01 and Bb01+, to serum was then analyzed by determining the bacterial titer after 1 or 2 h incubation of strains with mouse serum at 37°C. Our results showed that the titer of strain Bb01+ was significantly lower than that of strain Bb01 after 1 h incubation (Figure 3B, *P* < 0.05). Similar results were observed when *B. bronchiseptica* strains were incubated with serum for 2 h (Figure 3C, *P* < 0.001). Incubation with inactive serum or PBS did not decrease the titers of *B. bronchiseptica* strains, Bb01 and Bb01+ (Figures 3B,C). These results indicated that the specific integration of phage PHB09 significantly increased the sensitivity of *B. bronchiseptica* to mouse serum.

To investigate whether the specific integration of PHB09 affects the adhesion and invasion of *B. bronchiseptica*, same amount of Bb01 and Bb01+ were incubated with HEp-2 cells, respectively, at 37°C for 2 h. After washing out the unbound bacteria, the HEp-2 cells were lysed and the bacterial counts in the lysates were determined. Interestingly, as shown in Figure 3D, we found that the titer of Bb01+ was significantly lower than that of its parental strain, Bb01 (*P* < 0.01). Similarly, the titers of invaded *B. bronchiseptica* strains Bb01 and Bb01+ were determined individually after 4 h incubation with HEp-2 cells. We observed similar results that the titer of strain Bb01 was significantly higher than that of the strain Bb01+ (*P* < 0.01) (Figure 3E). We then employed RAW264.7 cells, a commonly used macrophage cell line, to evaluate the anti-phagocytic ability of strains Bb01 and Bb01+. Interestingly, we found that the titer of Bb01+ was significantly lower than that of Bb01 after 2 h incubation with RAW264.7 cells at 37°C (*P* < 0.05) (Figure 3F), indicating the integration of phage PHB09 decreased the anti-phagocytic ability of *B. bronchiseptica*.

### The Integration of Phage PHB09 Reduces the Virulence of *B. bronchiseptica in vivo*

**Figure 4:**
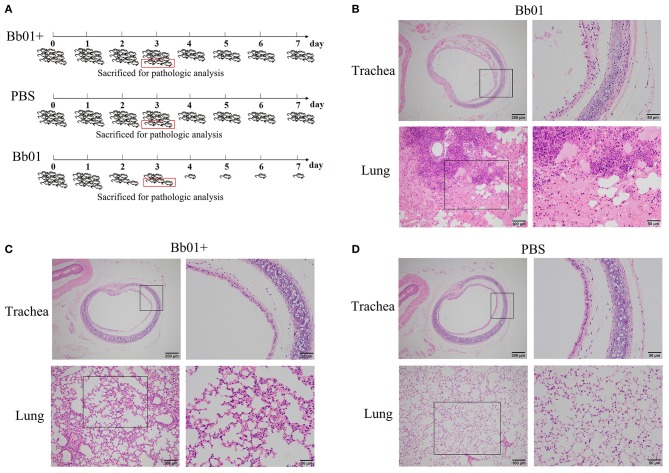
The virulence of *B. bronchiseptica* strains Bb01 and Bb01+ *in vivo*. **(A)** Mouse infection assay. Three groups of BALB/c mice were intranasally inoculated with Bb01, Bb01+, and PBS respectively and monitored daily for mortality. On day 3, three mice of each group were randomly selected and sacrificed for histopathologic analysis of tracheae (up panel) and lungs (low panel) of mice infected with Bb01 **(B)**, Bb01+ **(C)**, and PBS **(D)**. See results for the details. Survival was analyzed by Kaplan-Meier analysis with a log-rank test.

The virulence of *B. bronchiseptica* strains Bb01 and Bb01+ *in vivo* was investigated using BALB/c mouse model. We first determined the lethal ability of parent strain, Bb01, by intranasally inoculating three groups of mice (*n* = 3) with 20 μl PBS containing 2-fold serial dilutions of bacterial cells starting from 1.5 × 10^8^ CFU (Table S4). One of three mice died when 3.75 × 10^7^ CFU Bb01 was used, and all three mice died when the dose was increased to 1.5 × 10^8^ CFU. Therefore, 3.0 × 10^8^ CFU of *B. bronchiseptica* strains Bb01 and Bb01+ were used in the following challenge experiments, in which 27 mice were divided into three groups (*n* = 9) and inoculated with Bb01, Bb01+, and PBS respectively. As shown in Figure 4A, there was no death in Bb01+ challenged group. However, there were only 5 mice survived in Bb01 challenged group on day 3, but were severely sick. As expected, there was no death in the PBS control group (Figure 4A). Three mice from each group were randomly selected and sacrificed for pathological analysis, and the rest mice were monitored daily for mortality.

Since *B. bronchiseptica* is a respiratory pathogen, tracheae, and lungs were collected and used for pathological analysis. The representative figures were shown in Figures 4B–D, where no obvious lesions were observed in PBS control mice (Figure 4D). However, mice inoculated with *B. bronchiseptica* Bb01 showed severe lesions both in tracheae and lungs (Figure 4B). The tracheal mucosa was severely damaged and even disappeared, neutrophils infiltrated in the lamina propria, and the tracheal glands in the submucosa dilated (Figure 4B, up panel). The inherent structure of the lungs was damaged (Figure 4B, low panel), and multiple necrotic foci with bronchus as the center were observed. The bronchus in the necrotic foci was filled with exfoliated mucosal epithelial cells and inflammatory cells. The surrounding alveolar cavity was filled with necrotic tissue cells, neutrophils, monocytes, and blue-stained bacterial colonies. Most of the alveolar walls of the necrotic foci were hyperemia thickened, and the alveolar cavity was filled with serous exudate (Figure 4B, low panel). Interestingly, infection of *B. bronchiseptica* Bb01+ caused only minor pathological changes both in tracheae and lungs (Figure 4C). The tracheal mucosa was relatively intact, which was similar as observed in PBS group (Figures 4C,D, up panel). The inherent structure of the lungs was intact, and the alveolar walls were slightly thicker with capillary congestion and neutrophil infiltration compared to the PBS control group (Figures 4C,D, low panel). Taken together, these data indicated that the specific integration of phage PHB09 significantly decreased the virulence of *B. bronchiseptica* Bb01.

### The Phage Lysogenized Strain Is a Promising Vaccine Candidate Against *B. bronchiseptica*

**Figure 5:**
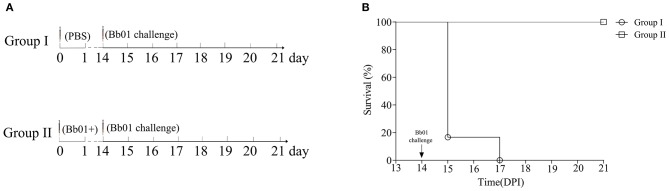
Phage PHB09 lysogenized *B. bronchiseptica* strain protects mice against lethal challenges with *B. bronchiseptica* Bb01. **(A)** Immunization scheme. Two groups of BALB/c mice were intranasally immunized with Bb01+ and PBS, respectively, and challenged with *B. bronchiseptica* strain Bb01 on day 14. **(B)** The survival curve of mice.

Due to the inherent ability to stimulate an excellent immune response, live attenuated vaccines are believed as the most effective vaccines. To test the potentiality of the avirulent *B. bronchiseptica* strain Bb01+ as a live attenuated vaccine, mice were immunized with strain Bb01+ and challenged virulent *B. bronchiseptica* strain Bb01 as shown in Figure 5A. The strain Bb01+ immunized group showed 100% protection against challenge with 3.0 × 10^8^ CFU *B. bronchiseptica* Bb01(Figure 5B). All mice in the control group, which was inoculated with PBS before challenge, died within 3 days post-challenge (Figure 5). The survival mice were sacrificed on 22-day post immunization (DPI) for histopathological analysis of lung and trachea. No significant histopathological changes were observed in both lung and trachea (data not shown). These results indicated that the prophage PHB09 bearing *B. bronchiseptica* strain Bb01+ could be a promising attenuated vaccine candidate.

## Discussion

It was demonstrated that phages are one of the driving forces of bacterial evolution and vice versa (Koskella and Brockhurst, 2014; Tao et al., 2018). The infection of lytic phages will finally lead to the burst of host cells to release progeny phages. Undoubtedly, bacteria can evolve resistance to phage infections through *de novo* mutation for survival, which might lead to the changes in bacterial traits (Buckling and Rainey, 2002). Due to the ability of horizontal gene transfer, temperate phages also affect the fitness and phenotype of their host bacteria (Argov et al., 2017; Howard-Varona et al., 2017). One attractive phenotype of pathogenic bacteria is the virulence, which determines the pathogenicity of the infectious disease. Particularly, the temperate phages can increase the virulence of the host bacteria by transmitting virulence genes, many of which were identified in phage genomes (Boyd, 2012).

The enhancement of bacterial virulence caused by temperate phage was frequently reported (Davies et al., 2016). However, temperate phage-mediated attenuation of virulence was rarely found. In this study, we reported a temperate phage PHB09 isolated from sewage water, and found that lysogeny of phage PHB09 significantly decreased the virulence of parental strain *B. bronchiseptica* Bb01 both *in vitro* (Figure 3) and *in vivo* (Figure 4). Sequence and morphological analysis indicated that it is a new phage and can be classified to *Siphoviridae* family. The genome of PHB09 encodes one tRNA gene and 59 putative ORFs (Figure 2A and Table S1), and no antibiotic resistance genes were identified (Table S2). Lysogeny related genes including integrase, which shows 40% identity to the integrase of *Pseudomonas* phage phiAH14a, and *cI* gene were identified.

The temperate phages integrate their genome into the host chromosome through either site-specific recombination (such as λ phage) or random transposition (such as Mu phage) (Campbell, 2003). We found the integration site of phage PHB09 in *B. bronchiseptica* genome is specific (Figures 2B,C) and locates in a gene coding a pilin protein, which showed the highest homology with *Oligella urethralis* pilin protein (Figure S1). Integration of phage PHB09 disrupts gene for the expression of pilin protein, which might lead to attenuation of *B. bronchiseptica* strain Bb01+. Pilin proteins form hair-like pili, which are found on the surfaces of bacteria, usually, are responsible for the adhesion to the host cells (Soto and Hultgren, 1999; Winther-Larsen et al., 2001). Many studies have identified pilin proteins as virulent factors of pathogenic bacteria, such as *E. coli* (Soto and Hultgren, 1999) and *Vibrio cholera* (Utada et al., 2014). Recently studies reported that pili of *Pseudomonas aeruginosa* are able to sense the extracellular signals, leading to the expression of hundreds of genes associated with pathogenicity (Persat et al., 2015). Therefore, it is possible that phage PHB09 mediated attenuation of *B. bronchiseptica* through disruption of the pilin gene. We tried to knock out the pili gene of *B. bronchiseptica* strain Bb01, but failed because it is very hard to edit *B. bronchiseptica* genome. Further studies are needed to confirm this possibility once the genome editing methods are available.

Furthermore, our results showed that immunization with the avirulent strain Bb01+ completely protected mice against lethal challenge with virulent *B. bronchiseptica*, indicating the vaccine potential of Bb01+. Most of the live attenuated vaccines were developed by mutating or deleting the virulence-associated gene(s) (Minor, 2015). Integration of a temperate phage disrupts gene for the expression of bacterial virulent gene, which will lead to the attenuation of the pathogen, and could be a new strategy to develop bacterial vaccines. However, it is well-known that prophages can switch to the lytic life cycle, in which the prophage DNA is excised from the host genome. Although such process often accompanied by lysis of the host cells, curing of prophages from host bacteria was reported in some bacteria (Euler et al., 2016; Aucouturier et al., 2018). Therefore, it is necessary to ensure there is no spontaneous release of prophage before using a lysogenized bacterium as a live attenuated vaccine. In case of prophage PHB09, we found that the induction of phage PHB09 always led to the lysis of *B. bronchiseptica* Bb01+. Therefore, there is little chance for *B. bronchiseptica* Bb01+ to reverse to the virulent state by release of prophage PHB09. Additionally, rather than a live attenuated vaccine, *B. bronchiseptica* Bb01+ can be used as an avirulent seed strain to develop inactivated vaccines.

Taken together, we report a temperate phage PHB09, which significantly decreases the virulence of *B. bronchiseptica*, most likely, due to the disruption of the pilin gene through specific integration. Our studies indicate the complicated roles of temperate phages in bacterial virulence other than the simple delivery of virulent genes. Our study also provides a new strategy to design bacterial vaccines.

## Data Availability Statement

The genome sequence of phage PHB09 and partial genome sequence of *B. bronchiseptica* Bb01 have been deposited in GenBank under accession numbers MN103401 and MN660071, respectively. The authors confirm all supporting data and protocols have been provided within the article or through Supplementary Data Files.

## Ethics Statement

This study was conducted in accordance with the *Guide for the Care and Use of Laboratory Animals of Hubei Province, China*. All animal experiments were performed according to the protocols approved by the Laboratory Animal Monitoring Committee of Huazhong Agricultural University, China.

## Author Contributions

YC, PT, and BW designed the experiments. YC and LY performed the experiments. YC, LY, JS, DY, CW, ES, and CG analyzed the data. YC and PT wrote the manuscript. HC, PT, YT, and BW directed the project.

### Conflict of Interest

The authors declare that the research was conducted in the absence of any commercial or financial relationships that could be construed as a potential conflict of interest.
