# Supplementary material for: Specific Integration of Temperate Phage Decreases the Pathogenicity of Host Bacteria
Source: Front Cell Infect Microbiol. 2020 Feb 4;10:14. doi: 10.3389/fcimb.2020.00014 (PMC7010805; doi:10.3389/fcimb.2020.00014)
Supplement: Supplementary file 1 [file Data_Sheet_1.docx]

**Supplementary Information for**

**Specific Integration of Temperate Phage Decreases the Pathogenicity of Host Bacteria**

Yibao Chen, Lan Yang, Dan Yang, Jiaoyang Song, Can Wang, Erchao Sun, Changqin Gu, Huanchun Chen, Yigang Tong^*^, Pan Tao^*^, Bin Wu^*^

**Supplementary information includes 4 tables and 1 figure.**

**Table S1. Phage PHB09 gene annotation**

| ORFs | Start | Stop | Length (bp) | Size (aa) | Function | Accession numbers | % identity | *E-*value |
| --- | --- | --- | --- | --- | --- | --- | --- | --- |
| 1 | 1012 | 77 | 936 | 311 | Integrase (endogenous virus) [Pseudomonas phage phiAH14a] | [AMW64461.1](https://www.ncbi.nlm.nih.gov/protein/AMW64461?report=genbank&log$=protalign&blast_rank=1&RID=CGX89XGW015) | 40% | 1.00E-72 |
| 2 | 1208 | 978 | 231 | 76 | Hypothetical protein |  |  |  |
| 3 | 1368 | 1252 | 117 | 38 | Hypothetical protein |  |  |  |
| 4 | 2837 | 1365 | 1473 | 490 | Transcriptional regulator [Bordetella phage vB_BbrM_PHB04] | [ATI15662.1](https://www.ncbi.nlm.nih.gov/protein/ATI15662?report=genbank&log$=protalign&blast_rank=1&RID=CGX89XGW015) | 31% | 7.00E-31 |
| 5 | 3841 | 2834 | 1008 | 335 | DNA-cytosine methyltransferase [Klebsiella phage 2b LV-2017] | [ARB15592.1](https://www.ncbi.nlm.nih.gov/protein/ARB15592?report=genbank&log$=protalign&blast_rank=2&RID=CGXM2N3U014) | 52% | 2.00E-108 |
| 6 | 4003 | 3845 | 159 | 52 | Hypothetical protein |  |  |  |
| 7 | 4857 | 4000 | 858 | 285 | DNA adenine methyltransferase [environmental Halophage eHP-8] | [AFH21726.1](https://www.ncbi.nlm.nih.gov/protein/AFH21726?report=genbank&log$=protalign&blast_rank=1&RID=EXYBURG2014) | 32% | 1.00E-32 |
| 8 | 5135 | 4854 | 282 | 93 | Hypothetical protein |  |  |  |
| 9 | 6061 | 5132 | 930 | 309 | Hypothetical protein |  |  |  |
| 10 | 7005 | 6058 | 948 | 315 | Putative transcriptional regulator [Salmonella phage LPST10] | [ARK07766.1](https://www.ncbi.nlm.nih.gov/protein/ARK07766?report=genbank&log$=protalign&blast_rank=2&RID=CGXZBVZW014) | 32% | 2.00E-30 |
| 11 | 7226 | 7008 | 219 | 72 | Hypothetical protein |  |  |  |
| 12 | 7360 | 7223 | 138 | 45 | Hypothetical protein |  |  |  |
| 13 | 7545 | 7904 | 360 | 119 | Hypothetical protein |  |  |  |
| 14 | 8899 | 8168 | 732 | 243 | CI protein [Escherichia phage JLK-2012] | [AFB75427.1](https://www.ncbi.nlm.nih.gov/protein/AFB75427?report=genbank&log$=protalign&blast_rank=3&RID=B43A4AN301R) | 58% | 8.00E-18 |
| 15 | 9000 | 9272 | 273 | 90 | Hypothetical protein |  |  |  |
| 16 | 9376 | 9906 | 531 | 176 | Hypothetical protein |  |  |  |
| 17 | 9903 | 10208 | 306 | 101 | Hypothetical protein |  |  |  |
| 18 | 10201 | 10503 | 303 | 100 | Hypothetical protein |  |  |  |
| 19 | 10500 | 10988 | 489 | 162 | Hypothetical protein |  |  |  |
| 20 | 10985 | 11842 | 858 | 285 | DNA replication protein O [Klebsiella phage 6 LV-2017] | [ARB15757.1](https://www.ncbi.nlm.nih.gov/protein/ARB15757?report=genbank&log$=protalign&blast_rank=1&RID=B42VYVSX015) | 41% | 4.00E-14 |
| 21 | 11862 | 12566 | 705 | 234 | Hypothetical protein |  |  |  |
| 22 | 12563 | 13051 | 489 | 162 | Hypothetical protein |  |  |  |
| 23 | 13048 | 13416 | 369 | 122 | Hypothetical protein |  |  |  |
| 24 | 13479 | 14090 | 612 | 203 | Hypothetical protein |  |  |  |
| 25 | 14294 | 15091 | 798 | 265 | Hypothetical protein |  |  |  |
| 26 | 15531 | 15253 | 279 | 92 | Hypothetical protein |  |  |  |
| 27 | 15784 | 15518 | 267 | 88 | BrnT family toxin [Bordetella bronchiseptica] | [WP_033451950.1](https://www.ncbi.nlm.nih.gov/protein/WP_033451950?report=genbank&log$=protalign&blast_rank=1&RID=B41MS75E014) | 99% | 7.00E-57 |
| 28 | 15987 | 16502 | 516 | 171 | Rha family transcriptional regulator [Achromobacter xylosoxidans] | [WP_104010591.1](https://www.ncbi.nlm.nih.gov/protein/WP_104010591?report=genbank&log$=protalign&blast_rank=1&RID=B41MS75E014) | 86% | 2.00E-104 |
| 29 | 16672 | 17004 | 333 | 110 | HNH nuclease [Vibrio phage 1.004.O._10N.261.54.A2] | [AUR81392.1](https://www.ncbi.nlm.nih.gov/protein/AUR81392?report=genbank&log$=protalign&blast_rank=1&RID=EXZCUR5M015) | 53% | 1.00E-05 |
| 30 | 17151 | 17615 | 465 | 154 | Terminase small subunit, partial [Vibrio phage 1.172.O._10N.261.52.F5] | [AUR92416.1](https://www.ncbi.nlm.nih.gov/protein/AUR92416.1?report=genbank&log$=protalign&blast_rank=1&RID=NGYMA9XX01R) | 47% | 6.00E-08 |
| 31 | 17619 | 19319 | 1701 | 566 | Putative large subunit terminase [Escherichia phage JLK-2012] | [AFB75457.1](https://www.ncbi.nlm.nih.gov/protein/AFB75457?report=genbank&log$=protalign&blast_rank=1&RID=CGTXF2P6015) | 56% | 0 |
| 32 | 19316 | 20662 | 1347 | 448 | Portal protein [Klebsiella phage KPP5665-2] | [ASX98619.1](https://www.ncbi.nlm.nih.gov/protein/ASX98619?report=genbank&log$=protalign&blast_rank=1&RID=CGUCU6R5014) | 31% | 5.00E-59 |
| 33 | 20628 | 21368 | 741 | 246 | Clp protease [Bacillus phage Tadhana] | [AUS03763.1](https://www.ncbi.nlm.nih.gov/protein/AUS03763?report=genbank&log$=protalign&blast_rank=2&RID=CGUCU6R5014) | 39% | 7.00E-37 |
| 34 | 21437 | 22675 | 1239 | 412 | Major capsid protein [Klebsiella phage 1 LV-2017] | [ARB15788.1](https://www.ncbi.nlm.nih.gov/protein/ARB15788?report=genbank&log$=protalign&blast_rank=1&RID=CGUCU6R5014) | 35% | 7.00E-72 |
| 35 | 22691 | 23125 | 435 | 144 | Hypothetical protein |  |  |  |
| 36 | 23128 | 23547 | 420 | 139 | Hypothetical protein |  |  |  |
| 37 | 23544 | 23882 | 339 | 112 | Head-tail adaptor protein [Acidovorax sp. Leaf160] | [WP_056661297.1](https://www.ncbi.nlm.nih.gov/protein/WP_056661297?report=genbank&log$=protalign&blast_rank=4&RID=CGV6X2F5014) | 39% | 3.00E-14 |
| 38 | 23875 | 24306 | 432 | 143 | Hypothetical protein |  |  |  |
| 39 | 24303 | 24653 | 351 | 116 | Hypothetical protein |  |  |  |
| 40 | 24718 | 25182 | 465 | 154 | Major tail subunit [Klebsiella phage 1 LV-2017] | [ARB15790.1](https://www.ncbi.nlm.nih.gov/protein/ARB15790?report=genbank&log$=protalign&blast_rank=1&RID=CGUUF68R014) | 32% | 3.00E-16 |
| 41 | 25189 | 25650 | 462 | 153 | Phage tail assembly chaperone [Escherichia phage phi467] | [CUW01215.1](https://www.ncbi.nlm.nih.gov/protein/CUW01215?report=genbank&log$=protalign&blast_rank=1&RID=CGUUF68R014) | 33% | 9.00E-11 |
| 42 | 25650 | 25943 | 294 | 97 | Hypothetical protein |  |  |  |
| 43 | 25983 | 29558 | 3576 | 1191 | Tail length tape-measure protein 1 [Acinetobacter phage Ab105-3phi] | [ALJ99019.1](https://www.ncbi.nlm.nih.gov/protein/ALJ99019?report=genbank&log$=protalign&blast_rank=1&RID=CGVWFBAZ014) | 35% | 9.00E-50 |
| 44 | 29562 | 29894 | 333 | 110 | Minor tail protein (endogenous virus) [Pseudomonas phage phiAH14a] | [AMW64537.1](https://www.ncbi.nlm.nih.gov/protein/AMW64537?report=genbank&log$=protalign&blast_rank=1&RID=CGVWFBAZ014) | 41% | 2.00E-31 |
| 45 | 29897 | 31468 | 1572 | 523 | Phage tail protein [Achromobacter sp. AONIH1] | [WP_103276057.1](https://www.ncbi.nlm.nih.gov/protein/WP_103276057?report=genbank&log$=protalign&blast_rank=8&RID=CGWBDN25014) | 43% | 1.00E-104 |
| 46 | 31470 | 31970 | 501 | 166 | Hypothetical protein |  |  |  |
| 47 | 31970 | 32716 | 747 | 248 | Minor tail protein (endogenous virus) [Pseudomonas phage phiAH14a] | [AMW64538.1](https://www.ncbi.nlm.nih.gov/protein/AMW64538?report=genbank&log$=protalign&blast_rank=1&RID=CGVWFBAZ014) | 39% | 5.00E-63 |
| 48 | 32720 | 33502 | 783 | 260 | Tail assembly protein K (endogenous virus) [Pseudomonas phage phiAH14a] | [AMW64541.1](https://www.ncbi.nlm.nih.gov/protein/AMW64541?report=genbank&log$=protalign&blast_rank=1&RID=CGVWFBAZ014) | 43% | 3.00E-68 |
| 49 | 33517 | 33876 | 360 | 119 | Hypothetical protein |  |  |  |
| 50 | 33964 | 34575 | 612 | 203 | Putative phage tail assembly protein [Edwardsiella phage eiDWF] | [ADV36465.1](https://www.ncbi.nlm.nih.gov/protein/ADV36465?report=genbank&log$=protalign&blast_rank=2&RID=CGWRG5YB014) | 54% | 2.00E-52 |
| 51 | 34572 | 38174 | 3603 | 1200 | Tail fiber protein (endogenous virus) [Pseudomonas phage phiAH14a] | [AMW64547.1](https://www.ncbi.nlm.nih.gov/protein/AMW64547?report=genbank&log$=protalign&blast_rank=1&RID=CGWRG5YB014) | 41% | 0 |
| 52 | 38179 | 38595 | 417 | 138 | Hypothetical protein |  |  |  |
| 53 | 38576 | 38788 | 213 | 70 | Hypothetical protein |  |  |  |
| 54 | 38820 | 39293 | 474 | 157 | Hypothetical protein |  |  |  |
| 55 | 39290 | 39838 | 549 | 182 | Hypothetical protein |  |  |  |
| 56 | 39838 | 40104 | 267 | 88 | Hypothetical protein |  |  |  |
| 57 | 40795 | 40265 | 531 | 176 | Hypothetical protein |  |  |  |
| 58 | 41251 | 40937 | 315 | 104 | Hypothetical protein |  |  |  |
| 59 | 41962 | 41303 | 660 | 219 | Hypothetical protein |  |  |  |

**Table S2. MIC distribution of *B. bronchiseptica* strains, Bb01 and Bb01+**

| Antibiotic | Bb01 | Bb01+ |
| --- | --- | --- |
| Ampicillin | <256 | < 256 |
| Ampicillin/Sulbactam | 64 | 64 |
| Tiamulin | 64 | 64 |
| Tilmicosin | 16 | 16 |
| Erythromycin | 4 | 4 |
| Ofloxacin | 2 | 2 |
| Ciprofloxa | 64 | 64 |
| Ceftiofur Sodium | < 256 | < 256 |
| Chloramphenicol | < 256 | < 256 |
| Sulfamethoxazole | < 256 | <256 |
| Gentamicin | 8 | 8 |
| Tetracycline | > 0.5 | > 0.5 |
| Trimethoprim | 64 | 64 |
| Polymyxin B | > 0.5 | > 0.5 |

**Table S3. Primers used for PHB09-lysogenized strains screening**

| Primers | Primer sequences (5’ to 3’) | PCR products (bp) |
| --- | --- | --- |
| 1F (cI) | CAAGCTGACGGTCGAAGAAATC | 352 |
| 1R (cI) | GACGTACCAGCCATTGCGAATTG |  |
| 2F (integrase) | GATTCGACTCGCAAGCAGTACGAG | 661 |
| 2R (integrase) | CGATGCCCTGTGATTCTGCATCTG |  |
| 3F (large subunit terminase): | CAAGGCGTCATTGAACGTCACAC | 1122 |
| 3R (large subunit terminase): | GAGTATGGGTTACTGATCAGCAAG |  |
| 4F | GTACGCATCGCCTGAACAG | 1030 |
| 4R | GACGTGCTAAAGATCGAG |  |
| 5F | GGTCAGCATGGTGTAGCTG | 1314 |
| 5R | GTGCGCAAGCTCATCGTG |  |

**Table S4. The survival rate of mice infected with *B. bronchiseptica* Bb01**

| Dose of bacteria Bb01 (CFU) | The survival rate of mice (mice surviving/ mice in group) |
| --- | --- |
| 3.75×10^7^ | 1/3 |
| 7.5×10^7^ | 1/3 |
| 1.5×10^8^ | 0/3 |

**Figure S1**





**Figure S1. Sequence analysis of *B. bronchiseptica* gene disrupted by phage PHB09 integration.** (A) The protein sequence of *B. bronchiseptica* gene (Query Sequence) was analyzed by Phyre2, a web server for analyzing protein structure and function. A homologous domain of PAK pilin protein from *Pseudomonas aeruginosa* (Template Sequence) was identified with a confidence value of 92.2% (45% coverage). (B) Protein-protein BLAST was used to identify the homologous pilin protein, which returns pilin protein of *Oligella urethralis* (GenBank accession No.SUA58787.1).
